# Ucma/GRP inhibits phosphate-induced vascular smooth muscle cell calcification via SMAD-dependent BMP signalling

**DOI:** 10.1038/s41598-018-23353-y

**Published:** 2018-03-21

**Authors:** Brecht A. Willems, Malgorzata Furmanik, Marjolein M. J. Caron, Martijn L. L. Chatrou, Dennis H. M. Kusters, Tim J. M. Welting, Michael Stock, Marta S. Rafael, Carla S. B. Viegas, Dina C. Simes, Cees Vermeer, Chris P. M. Reutelingsperger, Leon J. Schurgers

**Affiliations:** 1 Department of Biochemistry, School for Cardiovascular Diseases, Maastricht University, Maastricht, The Netherlands; 2 VitaK BV, Maastricht University, Maastricht, The Netherlands; 3 Department of Orthopedic Surgery, School for Public Health and Primary Care, Maastricht University, Maastricht, The Netherlands; 4 Department of Experimental Medicine I, Nikolaus-Fiebiger Centre of Molecular Medicine, University of Erlangen, Nuremberg, Germany; 5 Centre of Marine Sciences (CCMAR), University of Algarve, Faro, Portugal; 6 GenoGla Diagnostics, Centre of Marine Sciences (CCMAR), University of Algarve, Faro, Portugal

## Abstract

Vascular calcification (VC) is the process of deposition of calcium phosphate crystals in the blood vessel wall, with a central role for vascular smooth muscle cells (VSMCs). VC is highly prevalent in chronic kidney disease (CKD) patients and thought, in part, to be induced by phosphate imbalance. The molecular mechanisms that regulate VC are not fully known. Here we propose a novel role for the mineralisation regulator Ucma/GRP (Upper zone of growth plate and Cartilage Matrix Associated protein/Gla Rich Protein) in phosphate-induced VSMC calcification. We show that Ucma/GRP is present in calcified atherosclerotic plaques and highly expressed in calcifying VSMCs *in vitro*. VSMCs from Ucma/GRP^−/−^ mice showed increased mineralisation and expression of osteo/chondrogenic markers (BMP-2, Runx2, β-catenin, p-SMAD1/5/8, ALP, OCN), and decreased expression of mineralisation inhibitor MGP, suggesting that Ucma/GRP is an inhibitor of mineralisation. Using BMP signalling inhibitor noggin and SMAD1/5/8 signalling inhibitor dorsomorphin we showed that Ucma/GRP is involved in inhibiting the BMP-2-SMAD1/5/8 osteo/chondrogenic signalling pathway in VSMCs treated with elevated phosphate concentrations. Additionally, we showed for the first time evidence of a direct interaction between Ucma/GRP and BMP-2. These results demonstrate an important role of Ucma/GRP in regulating osteo/chondrogenic differentiation and phosphate-induced mineralisation of VSMCs.

## Introduction

Vascular calcification (VC) is a process of deposition of calcium phosphate crystals in the form of hydroxyapatite in the intima and media of the vessel wall^1,2^. The presence of VC reduces arterial wall elasticity and alters the hemodynamic profile, increasing the risk of cardiovascular events. VC is an independent risk factor predicting cardiovascular mortality and frequently measured as marker of atherosclerotic burden^3^. Nearly all patients suffering from cardiovascular disease have some degree of calcification, and in asymptomatic patients prevalence of coronary calcification corresponds with age. Amongst cardiovascular disease patients, approximately 60% of over 60-year-olds suffer from VC^3,4^. Chronic kidney disease (CKD) patients are known to be especially prone to VC. Mineral homeostasis dysregulation, uremic toxins and warfarin treatment are known to contribute to VC in dialysis patients^5,6^. Elevated phosphate in blood is thought to be one of the main inducers of VC in CKD^7,8^.

VC is an active process regulated by vascular smooth muscle cells (VSMCs). In response to cellular stress VSMCs undergo VC via several mechanisms: apoptosis, release of extracellular vesicles, loss of calcification inhibitors such as matrix Gla protein MGP; reviewed in Schurgers *et al*.^9^, ageing-related DNA damage and osteo/chondrogenic differentiation^10,11^.

In physiological conditions VSMCs exist in the vessel wall as contractile cells and regulate vascular tone. However, VSMCs are known to have a high degree of phenotypic plasticity^12^. In response to stress and injury, VSMCs lose expression of contractility-related genes such as SM22α, calponin (CNN1), and myosin light chain (MLC)^13–16^. When vascular injury is persistent, the phenotypic transition is dysregulated and VSMCs can undergo an unfavourable transdifferentiation into cells with characteristics of osteoblasts or chondrocytes^4,12,17–20^. This is termed osteo/chondrogenic differentiation. Many bone mineralisation-regulating proteins were found to be expressed in the calcifying blood vessel, such as BMP-2 Runx2, MGP, osteocalcin (OCN), osteopontin (OPN)^18,21,22^ amongst others and have been shown to play an active part in regulating VC. Additionally, calcifying VSMCs have been shown to release extracellular vesicles in a mechanism similar to release of matrix vesicles from chondrocytes^23^. However, the exact molecular mechanisms regulating VSMC osteo/chondrogenic transdifferentiation are unknown.

Ucma (Upper zone of growth plate and Cartilage Matrix Associated protein; also known as Gla Rich Protein - GRP) is a novel mineralisation inhibitor, which was first reported in cartilage^24,25^ and later in the vasculature^26,27^. Ucma/GRP-deficient mice did not develop a clear phenotype^28^, however *in vitro* studies revealed that Ucma/GRP regulates differentiation of chondrocytes and osteoblasts^29–31^. Using immunohistochemistry, Ucma/GRP was shown to be present at sites of VC. Moreover, when added exogenously, Ucma/GRP inhibited calcification of aortic rings *in vitro*^26,27,29^.

Based on the involvement of Ucma/GRP in differentiation of osteoblasts and its role in VC, we hypothesized that Ucma/GRP is involved in osteo/chondrogenic differentiation of VSMCs. To test our hypothesis we examined expression of Ucma/GRP during phosphate-induced calcification of VSMCs. We also examined osteo/chondrogenic gene expression and calcification of Ucma/GRP^−/−^ VSMCs exposed to elevated levels of inorganic phosphate. Our findings indicate that Ucma/GRP reduces VC by inhibiting osteo/chondrogenic VSMC transdifferentiation through a BMP-2-regulated pathway.

## Results

### Treatment of VSMCs *in vitro* with phosphate induces calcification

**Figure 1:**
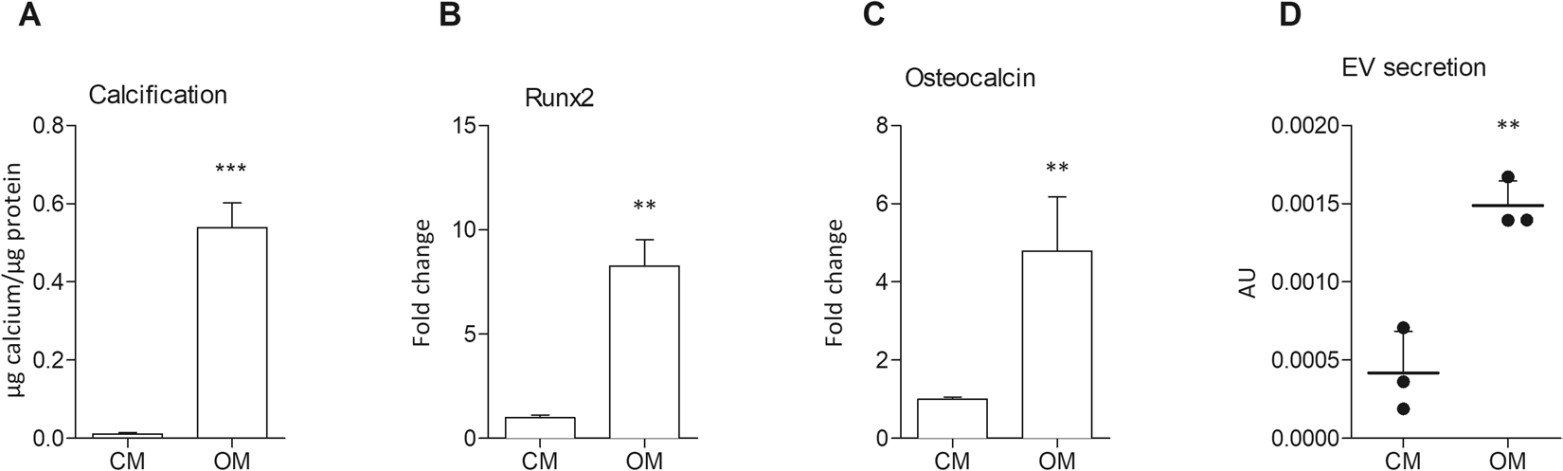
Increased phosphate concentrations induce calcification of mVSMCs *in vitro*. Cells were incubated with control medium (full growth medium, CM) or osteogenic medium (growth medium with 2.6 mM phosphate, OM) for 12 days. (**A**) Quantification of Ca content in mineralised mVSMC cultures, n = 4. (**B**,**C**) Calcification of mVSMCs was associated with increased expression of osteogenic genes Runx2 and osteocalcin measured by qPCR, n = 3. (**D**) Calcification of human VSMCs increased exosome secretion measured using a bead capture assay, n = 3. All graphs show mean + SD. Statistical significance was assessed using t-test. **p < 0.001–0.01, ***p < 0.001.

We first set out to examine calcification of primary mouse VSMCs *in vitro*. WT VSMCs were cultured in medium with elevated phosphate as described before^8,32^ (osteogenic medium, OM), which induced mineralisation *in vitro*, as observed by quantification of deposited calcium phosphate crystals (Fig. 1A). To control for spontaneous mineral precipitation on collagen, collagen-coated wells were incubated with OM in the absence of VSMCs. Under these a-cellular conditions, no calcification was observed (data not shown). To examine osteo/chondrogenic differentiation in mineralisation of VSMCs *in vitro*, Runx2 and Osteocalcin expression was measured using qPCR after 12 days of incubation with osteogenic medium (Fig. 1B and C). Expression of both markers was significantly increased, suggesting that the observed mineralisation is an active process involving osteo/chondrogenic differentiation. Moreover, treatment of human VSMC with osteogenic medium increased secretion of extracellular vesicles (Fig. 1D), which have recently been shown to mediate VC^23^.

### Ucma/GRP is expressed in atherosclerotic plaques of ApoE^−/−^ mice

**Figure 2:**
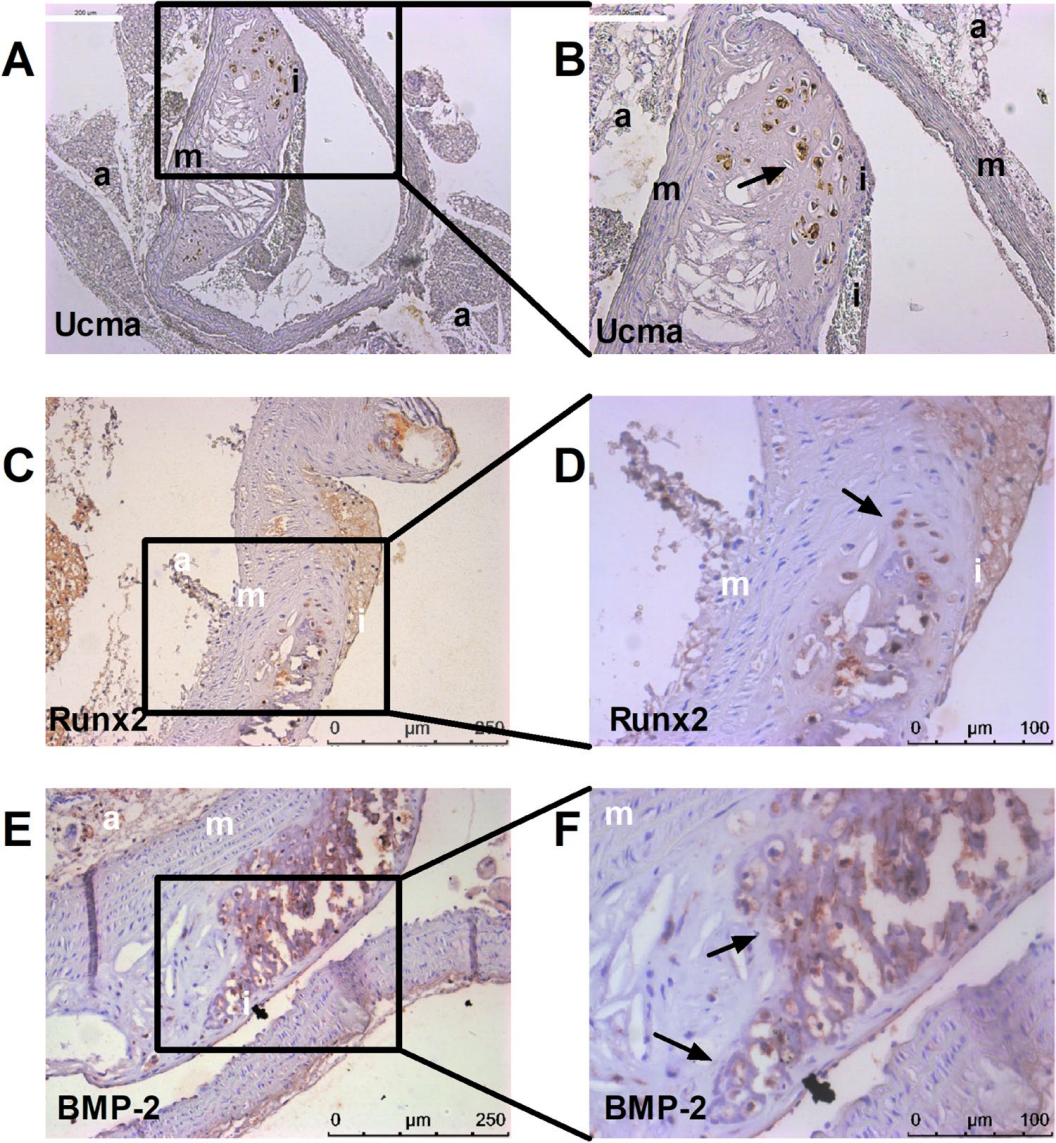
Ucma/GRP is expressed in atherosclerotic plaques. Sections of atherosclerotic plaques from ApoE^−/−^ mice fed a high fat diet for 12 weeks were stained for (**A**,**B**) Ucma/GRP, (**C**,**D**) BMP-2 and (**E**,**F**) Runx2. Arrows point to cells in the plaque with a chondrocyte-like morphology, which express both osteogenic markers and Ucma/GRP; a – adventitia, i – intima, m – media. Scale bars are 200 µm in A, 100 µm in B, D, F and 250 µm in C and E.

Ucma/GRP has previously been shown to be expressed in calcified human aortas and aortic valves^27^. Here we demonstrated that Ucma/GRP is expressed in advanced atherosclerotic lesions of ApoE^−/−^ mice. UcmaGRP was expressed specifically in atherosclerotic plaques (Fig. 2A and B) in cells which show similarities to chondrocyte morphology. As shown in Fig. 2C and D, these cells also expressed osteo/chondrogenic markers BMP-2 and Runx2. This suggests that Ucma/GRP is involved in osteo/chondrogenic differentiation and calcification of VSMCs in atherosclerotic plaques. Interestingly, not all cells with chondrocyte-like morphology visibly expressed Ucma/GRP.

### Ucma/GRP expression in VSMCs *in vitro* increases in response to osteogenic medium

**Figure 3:**
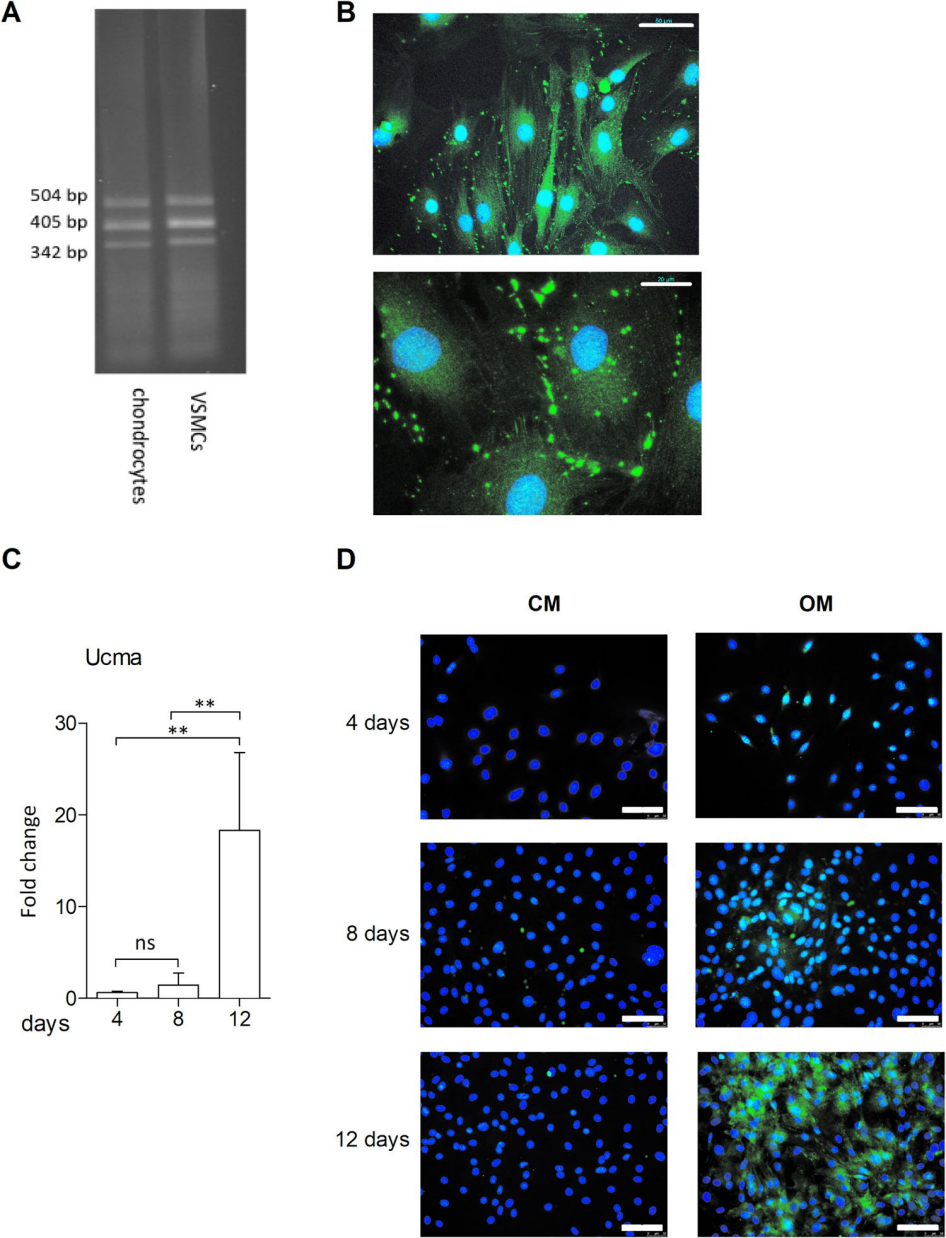
Ucma/GRP expression is increased in calcifying mVSMCs. Cells were incubated in growth medium (CM) or osteogenic medium (OM) containing 2.6 mM phosphate. (**A**) RT PCR of Ucma/GRP in mouse chondrocytes and VSMCs. Bands represent several transcript variants of Ucma/GRP. (**B**) Immunocytochemical staining of Ucma/GRP in mouse VSMCs. Scale bars are 50 µm (top) and 20 µm (bottom) (**C**) QPCR quantification of Ucma/GRP expression in calcifying mVSMCs. Graph shows mean + SD, n = 4. Statistical significance was assessed using ANOVA. **p between 0.001–0.01. (**D**) Immunocytochemical staining of Ucma/GRP in calcifying mVSMCs. Scale bars are 100 µm.

The presence of Ucma/GRP in murine VSMCs (mVSMCs) *in vitro* was confirmed using RT-PCR (Fig. 3A). Additionally, immunocytochemistry (Fig. 3B) has shown expression of Ucma/GRP in scattered cytoplasmic foci, which is consistent with its subcellular localization in other cell types^31^. Next, in order to examine whether Ucma/GRP has a role in mineralisation of VSMCs *in vitro*, expression was measured in response to treatment with osteogenic medium (Fig. 3C). Ucma/GRP expression, measured by qPCR, increased in a time-dependent manner with a 20-fold increase after 12 days of culture. Immunocytochemistry analysis confirmed this result and showed that control medium does not induce a similar increase (Fig. 3D).

### Ucma/GRP-deficient VSMCs are more prone to calcification in response to osteogenic medium

**Figure 4:**
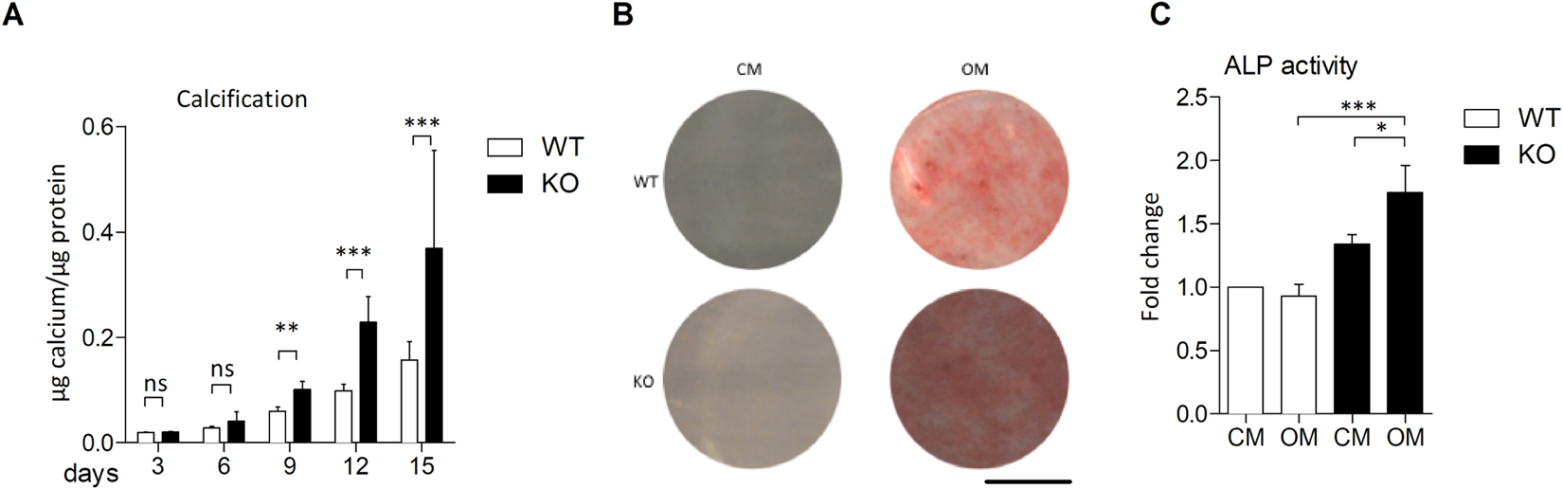
mVSMCs from Ucma/GRP^−/−^ mice show increased calcification. Cells from Ucma/GRP^−/−^ mice and WT counterparts were incubated with growth medium (CM) or osteogenic medium (OM) containing 2.6 mM phosphate. (**A**) Quantification of calcification over a 15 day period. Statistical significance was assessed using t-test, n = 3–5. (**B**) Alizarin red S staining of calcium deposited by mVSMCs after 12 days. Scale bar is 80 mm. (**C**) Alkaline phosphatase (ALP) in mVSMCs treated with osteogenic medium or control medium for 12 days. Statistical significance was assessed using ANOVA, n = 3. All graphs show mean + SD. *p between 0.05–0.01, **p between 0.001–0.01, ***p < 0.001.

Aortas were harvested from Ucma/GRP^−/−^ mice (KO) and wild type (WT) counterparts generated as previously described^28^. The knock-out of Ucma/GRP was confirmed by immunohistochemical staining of the epiphyseal plate (Supplemental Fig. 1A). VSMCs were isolated and their identity was verified by immmunocytochemical staining for VSMC markers (αSMA, CNN1, p-MLC) (Supplemental Fig. 1B). We and others^26,27^ have shown that Ucma/GRP is upregulated in response to calcification and that its expression is confined to osteo/chondrogenic cells in atherosclerotic plaques (Figs 2A and 3A,B). Therefore, we hypothesized that Ucma/GRP is involved in controlling osteo/chondrogenic differentiation of VSMCs and consequently calcification. To test this, WT and Ucma/GRP^−/−^ VSMCs were incubated in osteogenic medium over a time span of 15 days (Fig. 4A and B). Both WT and Ucma/GRP^−/−^ VSMCs calcified in response to osteogenic medium, with calcification becoming detectable after 6 days. However, Ucma/GRP^−/−^ VSMCs calcified approximately twice as much as WT cells after 9, 12 and 15 days. Neither Ucma/GRP^−/−^ nor WT VSMCs calcified in control medium (Supplemental Fig. 1C). Ucma/GRP knock-down in WT cells using siRNA resulted in significantly increased calcification compared to cells transfected with a scramble control (Supplemental Fig. 1D), confirming that the observed effects are specific to Ucma/GRP. In response to osteogenic medium alkaline phosphatase (ALP) activity was increased in Ucma/GRP^−/−^ cells but not in WT cells, suggesting increased osteo/chondrogenic potential of Ucma/GRP^−/−^ cells (Fig. 4C).

### Ucma/GRP deficiency results in increased osteo/chondrogenic gene expression

**Figure 5:**
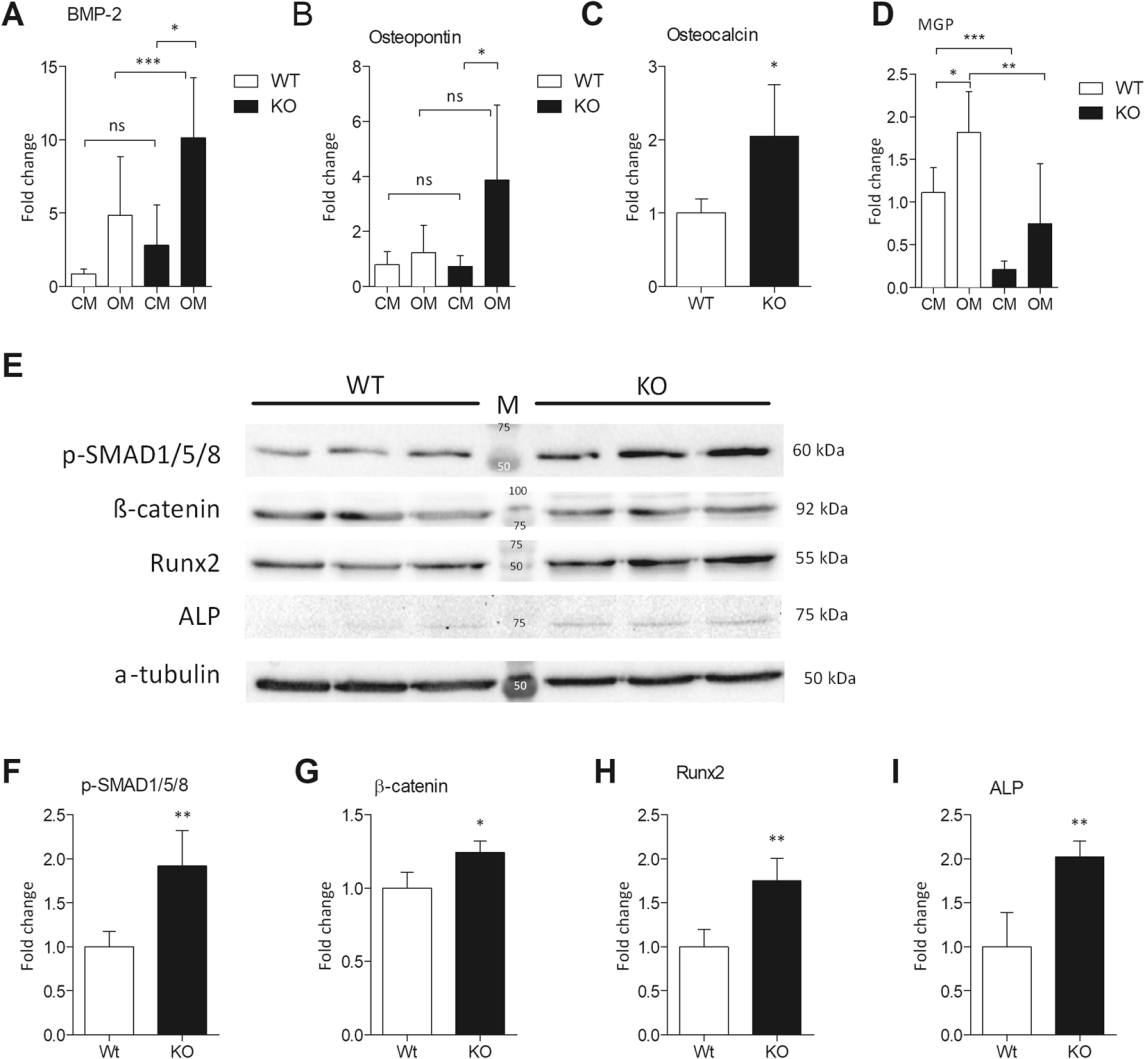
Ucma/GRP^−/−^ mVSMCs show increased expression of osteo/chondrogenic markers. Cells were treated with control (CM) or osteogenic medium (OM, with 2.6 mM phosphate) for 12 days. Panels (A–D) qPCR analysis of osteo/chondrogenic gene expression, n = 4–7. Statistical significance was assessed using t-test (Osteocalcin) or ANOVA (**E**) Western blotting analysis of osteo/chondrogenic markers. Both WT and KO cells were treated with OM. Figure shows full western blots obtained by cutting of a full-sized membrane prior to antibody incubations. Numbers in the middle show molecular weights of the protein standard. Numbers on the right show the expected molecular weights of the proteins. Panels F–I quantification of western blotting results from panel E, n = 3. Statistical significance was assessed using t-test. All graphs show mean + SD. *p between 0.05–0.01, **p between 0.001–0.01.

Since osteo/chondrogenic differentiation is an important mechanism regulating VC and expression of osteo/chondrogenic markers is found in atherosclerotic plaques, we set out to examine whether there were any differences in osteo/chondrogenic marker expression between WT and Ucma/GRP^−/−^ VSMCs. We observed that Ucma/GRP^−/−^ cells expressed increased levels of BMP-2 mRNA in baseline conditions (CM, Fig. 5A). Additionally, when challenged with osteogenic medium, Ucma/GRP^−/−^ VSMCs expressed higher levels of OPN, Osteocalcin and BMP-2, compared to WT VSMCs (Fig. 5A,B and C). MGP mRNA expression was significantly downregulated in Ucma/GRP^−/−^ compared to WT VSMCs, both in baseline and osteogenic conditions, in line with the increased calcification potential of these cells (Fig. 5D). Upon treatment with osteogenic medium MGP expression increased, both in WT and Ucma/GRP^−/−^ VSMCs. Western blot analysis demonstrated an increase in β-catenin and phosphorylated SMAD1/5/8 expression, suggesting the involvement of SMAD signalling pathways (Fig. 5E,F,G) known to be involved in osteoblast mineralization and VC. Additionally, expression of Runx2 and ALP was higher in Ucma/GRP^−/−^ cells compared to WT (Fig. 5E,H and I). Importantly, we also observed that Ucma/GRP^−/−^ VSMCs displayed reduced expression levels of contractile phenotype marker αSMA compared to WT VSMCs (Supplemental Fig. 1E). Taken together these results show that Ucma/GRP^−/−^ VSMCs undergo increased osteo/chondrogenic differentiation, compared to WT VSMCs, both at baseline as well as in osteogenic conditions.

### The osteo/chondrogenic phenotype in Ucma/GRP^−/−^ VSMCs is induced by lack of inhibition of a BMP-2/4-p-SMAD1/5/8-β-catenin dependent-pathway

**Figure 6:**
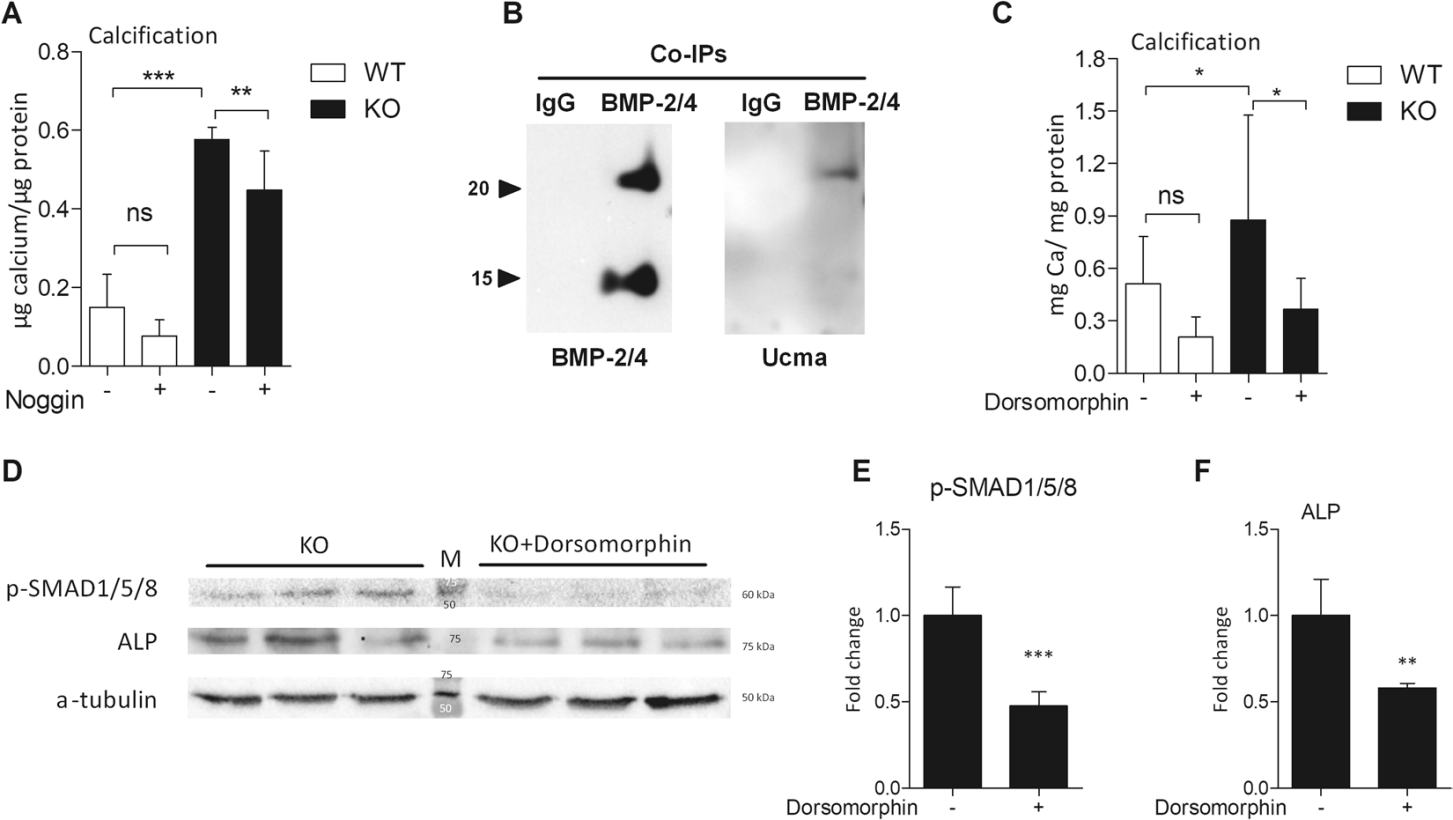
Ucma/GRP is involved in suppressing osteo/chondrogenic differentiation by interacting with BMP-2. (**A**) Quantification of calcification deposited by mVSMCs incubated with 100 ng/ml noggin in the presence of osteogenic medium (2.6 mM phosphate) for 12 days, n = 6. Statistical significance was assessed using ANOVA. (**B**) Conditioned media from hVSMCs treated with 500 ng/ml of BMP-2 for 24 h was used to capture BMP-2-containing protein complex by Co-immunoprecipitation (Co-IP) with a BMP2/4 antibody, and the presence of BMP2 and GRP in the eluted complex was detected by western blot. Two bands (25 kDa and 15 kDa) correspond to the dimeric and monomeric BMP-2/4. Representative image of 3 independent experiments. (**C**) Quantification of calcification deposited by mVSMCs from Ucma/GRP KO and WT mice, n = 8. Cells were incubated with 2 µM dorsomorphin in osteogenic medium (OM) for 12 days. Statistical significance was assessed using ANOVA. (**D**) Western blotting analysis of phosphorylated SMAD 1/5/8 and ALP and in Ucma/GRP^−/−^ mVSMCs treated with 2 µM dorsomorphin in osteogenic medium (OM) for 12 days. Figure shows full western blots obtained by cutting of a full-sized membrane prior to antibody incubations. Numbers in the middle show molecular weights of the protein standard. Numbers on the right show the expected molecular weights of the proteins. (**E**,**F**) Quantification of Western blots from panel C, n = 3. Statistical significance was assessed using t-test. All graphs show mean + SD. *p between 0.05–0.01, **p between 0.001–0.01, ***p < 0.001.

BMP-2 is a key modulator in the development of VC^19,21^. Since we observed an increase in BMP-2 expression and its downstream targets p-SMAD1/5/8, we investigated the involvement of BMP signalling in calcification of Ucma/GRP^−/−^ VSMCs. We blocked BMP-2 and BMP-4 signalling using noggin and treated VSMCs with osteogenic medium (Fig. 6A). Noggin treatment decreased calcification of both WT and Ucma/GRP^−/−^ VSMCs. Additionally, to investigate whether there is an interaction between BMP-2/4 and Ucma/GRP we carried out co-immunoprecipitation using media from hVSMCs treated with BMP-2. BMP-2/4-containing protein complexes were captured with a BMP-2/4 antibody and analysed using western blotting for Ucma/GRP. The results show clearly the presence of Ucma/GRP (Fig. 6B), strongly pointing to a functional BMP-2/4-Ucma/GRP interaction. To examine whether there is a direct interaction with BMP-2, we carried out solid phase binding assays by coating ELISA plates with carboxylated Ucma/GRP (cUcma), uncarboxylated (ucUcma), BMP-2 and MGP (positive control) and incubating with BMP-2 in the liquid phase. Detection of BMP-2 with an anti-BMP-2/4 antibody showed that BMP-2 bound to Ucma/GRP, specifically to the carboxylated Ucma/GRP form (Supplemental Fig. 2A).

Further to that, to investigate the involvement of specific downstream targets of BMP-2 we treated cells with dorsomorphin, an inhibitor of SMAD1/5/8 activation (AMPK inhibitor). Dorsomorphin decreased calcification of WT VSMCS and restored levels of calcification in Ucma/GRP^−/−^ cells to the levels observed in WT VSMCs (Fig. 6C). The reduction in calcification was reflected by a decrease in p-SMAD1/5/8 and ALP expression (Fig. 6D,E,F). Taken together, these results suggest that increased osteo/chondrogenic and calcification potential of Ucma/GRP^−/−^ VSMCs is due to impaired inhibition of SMAD-dependent BMP signalling.

As Erk signalling has been known to also activate SMAD signalling^33^, we investigated whether inhibiting Erk phosphorylation has an effect on calcification of Ucma/GRP^−/−^ VSMCs. Treatment of WT and Ucma/GRP^−/−^ VSMCs with Erk phosphorylation inhibitor U0126 did not change calcification levels of cells of either genotype, suggesting that Erk phosphorylation and Ucma/GRP are not interacting to inhibit calcification of VSMC cultures (Supplemental Fig. 2B).

## Discussion

VC is an important predictor of cardiovascular mortality. In particular, CKD patients have been shown to be at an increased risk of VC^5,34^. However, the mechanisms through which VC develops remain incompletely understood. In the present study we demonstrate that Ucma/GRP plays a role in the attenuation of osteo/chondrogenic differentiation of VSMCs through a pathway involving SMAD-dependent BMP signalling. Osteo/chondrogenic switching is accelerated in the absence of Ucma/GRP and this results in enhanced calcification. We also provide evidence that high phosphate concentrations, which is a common finding in CKD patients^7^, induce upregulation of Ucma/GRP in VSMCs. Our findings reveal a novel pathway through which Ucma/GRP inhibits phosphate-induced SMAD signalling and osteo/chondrogenic differentiation, thereby reducing the propensity for calcification of the vessel wall.

Ucma/GRP was previously shown to inhibit maturation of chondrocytes towards hypertrophic cells and osteogenic differentiation of osteoblasts^29^. Ucma/GRP is known to be present in low levels in calcified aortic valves and in the media. Ucma/GRP inhibits calcification of aortic rings cultured in calcifying medium, when added exogenously to cultures, this was accompanied by an increase in αSMA expression and a decrease of osteopontin expression^27^. In our study we confirmed the presence of Ucma/GRP in the vasculature and for the first time reveal that Ucma/GRP expression is markedly increased in osteo/chondrogenic cells in atherosclerotic plaques of ApoE^−/−^ mice, which implicates its role in this vascular pathology. CKD patients show accelerated atherosclerosis, which suggests that our *in vivo* findings are relevant for calcification in CKD-related atherosclerosis^35,36^.

BMP-2 is a potent inducer of calcification and regulator of osteo/chondrogenic differentiation of VSMCs, known to cause phosphate influx into cells^21^. BMP-2 acts by binding its cell surface receptors which transduce the signal to the cytoplasm by phosphorylating pathway-restricted SMADs (SMAD1 and SMAD5) for BMPs^37^. This leads to heterodimerization of pathway-restricted SMADs with SMAD4, a common-mediator SMAD, and translocation of the complex to the nucleus, where it binds directly to DNA^38^. As a result, changes in gene expression occur, such as upregulation of Runx2, OCN and ALP. Additionally, BMP-2 has been shown to induce accumulation of β-catenin^39^. β-catenin has been demonstrated to activate Runx2 expression in the context of phosphate-induced VSMC calcification^40^. BMP and Wnt pathways have been shown to be co-activated in calcifying VSMCs^41^, where SMAD1 and β-catenin interacted and regulated gene expression together. Additionally, dorsomorphin homologue 1 has been recently shown to inhibit phosphate-induced osteogenic differentiation of hVSMCs by inhibiting BMP-2^42^. Our data are in line with these findings, as we demonstrate increased SMAD1/5/8 phosphorylation and expression of β-catenin, Runx2 and their downstream targets ALP, OCN and OPN in phosphate-treated Ucma/GRP^−/−^ VSMCs. Here we demonstrate for the first time that Ucma/GRP interacts with this BMP-SMAD signalling pathway by binding BMP-2 and that inhibiting BMP-2/-4 signalling with noggin and SMAD1/5/8 phosphorylation with dorsomorphin decreased calcification of VSMCs and osteo/chondrogenic gene expression. Interestingly, previous reports show that externally added BMP-2 decreased Ucma/GRP expression in chondrocytes^29^, suggesting an interaction between BMP-2 signalling and Ucma/GRP. In addition to that, in osteoblasts Ucma/GRP is under direct transcriptional control of Runx2 and Osterix^30^, however this has not been investigated in VSMCs.

In our study we confirm that high levels of phosphate induce hallmarks of osteogenic differentiation in VSMCs, such as osteo/chondrogenic gene expression and extracellular vesicle release, and that Ucma/GRP expression is increased in VSMCs treated with osteogenic medium *in vitro*. This, together with the presence of Ucma/GRP in advanced atherosclerotic lesions, suggests that calcifying cells increase the expression of this inhibitor in a bid to protect themselves from adverse phenotype changes. This has been shown to happen with MGP, another vitamin K-dependent calcification inhibitor, by us in the present study and others^43^. Additionally, insufficient carboxylation of MGP due to warfarin administration is one of the factors contributing to VC in CKD^44^. Uncarboxylated Ucma/GRP, similar to uncarboxylated MGP, could contribute to these processes. Ucma/GRP is a vitamin K-dependent protein, as it undergoes posttranslational carboxylation^45^. It has been previously shown that uncarboxylated Ucma/GRP accumulates at sites of pathological calcification in human aortic valves and media and only the carboxylated form of Ucma/GRP was able to inhibit calcification^27^. Our results are in line with this, as we show that carboxylated Ucma/GRP has high affinity to BMP-2, whereas uncarboxylated Ucma/GRP binds BMP-2 only weakly. However, we have not investigated the carboxylation status of Ucma/GRP in the atherosclerotic plaques which we examined and this warrants further investigation.

MGP is known to inhibit VC by binding mineral and interacting with BMP-2, preventing it from binding its receptor^7,9,23^. It is therefore tempting to speculate that vitamin K-dependent inhibitors complement each other with respect to regulating VC: calcium-induced calcification is regulated by MGP and whereas phosphate-induced calcification is regulated by Ucma/GRP. This is further supported by previous studies showing that phosphate increases osteo/chondrogenic differentiation of VSMC *in vitro*, whereas this was not demonstrated for calcium yet^8,12,46^.

**Figure 7:**
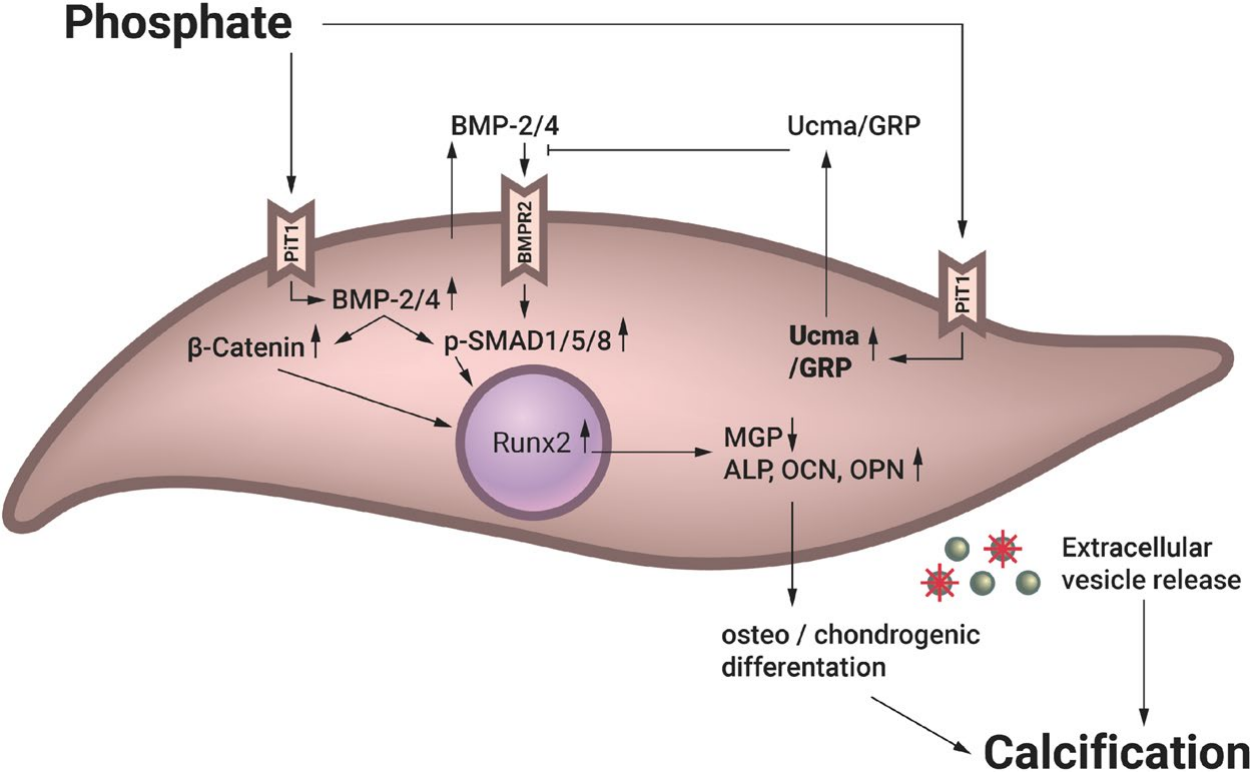
Proposed model of the protective effect of Ucma/GRP in calcifying mVSMCs. Ucma/GRP expression is increased as a negative feedback loop mechanism when extracellular phosphate is elevated. Ucma/GRP prevents activation of phosphate-induced BMP-2-SMAD signalling in calcifying VSMCs, acting as an inhibitor of signalling cascades that lead to calcification. OCN – osteocalcin, OPN – osteopontin, ALP – alkaline phosphatase, MGP – matrix Gla protein.

Based on our observations, we propose a model of how Ucma/GRP inhibits VC (Fig. 7). When VSMCs are exposed to elevated phosphate concentrations in the extracellular environment, as is the case in CKD patients, osteo/chondrogenic differentiation (as evidenced by increased Runx2, β-catenin, osteocalcin, osteopontin and ALP expression) is initiated via increased BMP-2-SMAD signalling. We propose that Ucma/GRP can ameliorate the effect of increased phosphate by inhibiting this signalling pathway and thus reduce calcification of the vessel wall.

Taken together, we demonstrate that Ucma/GRP, a member of a range of inherent calcification inhibitors present in the vasculature^47^, plays an important role in regulating osteo/chondrogenic differentiation of VSMCs. Our findings have implications for vascular pathologies which lead to VC and represent an advancement in our knowledge about the steps resulting in VC. One of the main goals in the treatment of patients with CKD is to reduce the substantially increased risk of cardiovascular comorbidity and mortality, improving both quality and length of life. The increase in CKD stage and cardiovascular risk is accompanied by increased phosphate levels. As shown by our results, the phosphate-Ucma/GRP-BMP axis holds promise for novel interventions. Pharmacological manipulation of Ucma/GRP levels could represent a novel therapeutic strategy to prevent VC.

## Methods

### Experimental animals

Ucma/GRP^−/−^ C57BL\6 mice were generated and kindly provided by Dr. M. Stock, University of Erlangen, Germany, with the approval of the local ethics authorities (University of Erlangen-Nuremberg and the Government of Mittelfranken, Ansbach, Germany) and according to the regulations of the animal facilities in Germany^28^. Mice were maintained in a specific pathogen-free environment with free access to water and western type diet (Arie Block, Woerden, the Netherlands) and sacrificed by portal vein puncture after 12 weeks. Aortas were harvested for immunohistochemistry and VSMC isolation.

### Immunohistochemistry

Aortic sections of atherosclerotic ApoE^−/−^ mice from a previous study were used^48^. Sequential 4 µm paraffin sections were cut from the aortic arch and used for immunohistochemical staining as previously described^48^. The following antibodies were used: anti- BMP-2 (Santa Cruz, sc-6895), anti-Runx2 (M70, Santa Cruz, sc-10758) anti-total Ucma/GRP antibody (Cterm-GRP, GenoGla Diagnostics, Faro, Portugal), HRP conjugated secondary antibodies and DAB or Nova-RED substrate (Vector Labs, Amsterdam, the Netherlands). Sections were counterstained with haematoxylin (Klinipath, Duiven, the Netherlands) and mounted in entellan (Merck, Amsterdam, the Netherlands).

### VSMC isolation and culture

Endothelium and adventitia were removed from aortas of Ucma/GRP^−/−^ and WT littermates. Tissue was dissected into ± 5 mm² pieces and digested with 3 mg/ml collagenase (Sigma, Zwijndrecht, the Netherlands) and 1 mg/ml elastase (Sigma) for 4 hours at 37 °C in DMEM (Gibco, Bleiswijk, the Netherlands). The cells were washed, resuspended in growth medium (DMEM with 10% FCS, Gibco, 100 U/ml penicillin and 100 mg/ml streptomycin, Gibco) and transferred to culture dishes. Success of the isolation was determined by immunofluorescent staining for αSMA, SM22α, phosphorylated myosin light chain 2 (method below) and western blotting for αSMA. Cells at passage 4–12 were used for experiments. Cells were maintained in culture medium and passaged when 90% confluent. Cell cultures were not supplemented with additional vitamin K. The following inhibitors were added to media for treatments: 2 µM of U0126 (Merck, Amsterdam, the Netherlands), 100 ng/ml noggin (R&D systems, Abingdon, United Kingdom), 2 µM dorsomorphin dihydrochloride (sc-361173, Santa Cruz). All inhibitors were added to cells for the full duration of the experiment, as indicated in figure legends. Medium was refreshed every 2–3 days.

Human primary VSMCs (hVSMCs) were used for exosome quantification assay and co-immunoprecipitation. They were maintained in M199 with 20% FCS, 100 U/ml penicillin and 100 mg/ml streptomycin (Gibco).

### Immunofluorescence

Cells were fixed with 4% PFA, permeablilised with 0.1% Triton X-100, blocked in 1% BSA/PBS and incubated with primary antibodies (Calponin, Abcam, ab46794; αSMA,Sigma; phosphorylated myosin light chain 2, Cell Signalling, 3675S and anti-total Ucma/GRP antibody (Cterm-GRP, GenoGla Diagnostics, Faro, Portugal). The following secondary antibodies were used: anti-rabbit-FITC (Dako, F0205) and anti-mouse-FITC (Dako, F0232). Nuclei were stained with DAPI (Sigma–Aldrich). Cells were mounted in a glycerol DABCO mounting medium. Pictures were taken with a Leica DM2000 widefield microscope (Leica Microsystems).

### Calcification assays

4 × 10^4^ cells were seeded per well of a 12-well plate. After 24 hours medium was changed to osteogenic medium (growth medium +2.6 mM PO_4_^3−^, OM) or growth medium (control medium, CM). Quantification of deposited calcium was carried out using a calcium determination kit (Randox, London, United Kingdom) according to the manufacturer’s instruction, after solubilising mineral deposits in 0.1 M HCl. Calcium measurements were normalised to protein content using micro BCA assay (Thermo Scientific, Bleiswijk, the Netherlands). Calcification was additionally visualised using Alizarin Red S staining adapted from Gregory *et al*.^49^.

### RNA isolation and quantitative real-time PCR

Total RNA was isolated using TRI Reagent (Sigma). RNA concentration and quality were determined using a Nanodrop 2000 spectrophotometer (Thermo Scientific, Bleiswijk, the Netherlands) and agarose-denaturing gel electrophoresis. 1 µg of total RNA was treated with DNAse I (Promega, Leiden, the Netherlands) and reverse transcribed using M-MLV reverse transcriptase (Invitrogen, Bleiswijk, the Netherlands), an oligo dT adapter (Eurogentec, Maastricht, the Netherlands) and RNAse Out (Invitrogen, Bleiswijk, the Netherlands). Real-time qPCR was performed using the Quantitect SYBR green PCR kit (Qiagen, Venlo, the Netherlands) in a LightCycler 480 II (Roche, Woerden, the Netherlands) with 50 ng of cDNA and 0.5 µM of each primer. The following primers were used BMP-2 (F) GTG-CTT-CTT-AGA-CGG-ACT-GCG-GTC-TCC-TA, BMP-2 (R) GCC-TGA-GTG-CCT-GCG-GTA-CAG-ATC-TAG, Runx2 (F) GAC-GAG-GCA-AGA-GTT-TCA-CC, Runx2 (R) GGA-CCG-TCC-ACT-GTC-ACT-TT, HPRT1 (F) AGC-CAA-ATA-CAA-AGC-CTA-AGA-TGA-GCG, HPRT1 (R) GAA-ATG-TCA-GTT-GCT-GCG-TCC-CCA-GA, MGP (F) ACA-CAG-AGG-CAG-ACT-CAC-AGG-ACA-CCC, MGP (R) CTG-AGG-GGA-CAT-AAA-GGT-GTT-GGC-AT, OCN (F) AAG-CAG-GAG-GGC-AAT-AAG-GT, OCN (R) TTT-GTA-GGC-GGT-CTT-CAA-GC, OPN (F) TGA-CCA-CAT-GGA-CGA-CGA-TGA-TGA-CGA-TGA, OPN (R) GGG-ACG-ATT-GGA-GTG-AAA-GTG-TCT-GCT-TGT. Fluorescence curves were analyzed with LightCycler 480 Software (Version 1.5) and relative quantification was performed with the 2^−ΔΔCt^ method. Ucma/GRP was detected as described previously^45^. All samples were assayed in triplicate.

### Quantification of exosome release

Exosomes were quantified using a bead-capture assay as described before^23^. Briefly, anti-human CD63 antibody (556019, BD Bioscience) was immobilized on aldehyde-sulfate functionalized beads (Invitrogen). Human primary VSMC culture media was incubated with anti-CD63-coated beads on a shaker overnight at 4 °C. VSMCs were stained with Hoechst 33342 (Thermo Fisher) and counted using a Cytation 3 live-cell imager. Beads were washed and incubated with anti-CD81-PE antibodies for 1 h at room temperature. Then beads were washed with PBS with 2% BSA and analyzed by flow cytometry (Accuri C6, BD). Arbitrary units (AU) were calculated as mean fluorescence units times percentage of positive beads and normalized to the number of cells.

### Western blotting

VSMCs were lysed in 0.1 M Tris pH 8.1, 0.15 M NaCl, 1% triton x-100 0.2 mM NaVO_3_ and 1:50 protease inhibitor cocktail (Sigma). Protein concentrations were determined using DC protein assay (Bio-Rad, Veenendaal, the Netherlands) and lysates were separated on Any kD Mini-PROTEAN TGX Precast Protein Gels (BioRad). Samples were transferred to a PVDF or nitrocellulose membrane (BioRad) and incubated overnight with anti-αSMA (A2547, Sigma), anti-calponin (ab46794, Abcam), anti-ALP (R&D Systems, AF290), anti-Runx2 (MBL, D130-3), anti-β-catenin (BD, 610153), anti-p-SMAD1/5/8 (Cell Signalling, 12820P) and α-tubulin (Sigma, T6074) antibodies. Protein was then detected using HRP-conjugated secondary antibodies (anti-mouse: p0447, Dako; anti-rabbit: 7074S, Cell Signalling, anti-goat: P0449, Dako) and visualized by enhanced chemiluminescence (Pierce ECL Western Blotting Substrate, ThermoFisher Scientific).

### Co-immunoprecipitation

Co-IP assays were performed using conditioned media from hVSMCs cultured in the presence of 500 ng/ml of recombinant BMP-2 (Preprotech) for 24 h in M199 with 10% FBS, and the Dynabeads Co-immunoprecipitation Kit (Novex, Life Technologies), according to manufacturer’s recommendations. The BMP-2/4 monoclonal (Santa Cruz Biotechnology, sc-137087) and negative IgG antibodies were used as capture antibodies in the Co-IP reactions, and Western blot analysis of the eluted proteins were performed using CTerm-Ucma/GRP (GenoGla Diagnostics, Faro, Portugal) and BMP-2/4 antibodies as described above.

### Alkaline phosphatase activity

Cells were lysed in 1% Triton X-100 in PBS, subjected to 2 freeze-thaw cycles and centrifuged at 13000 g for 5 minutes. ALP activity in the supernatants was measured at 405 nm using 4-Nitrophenyl phosphate disodium salt hexahydrate (Sigma) as substrate. Enzyme activity (U) was normalised to protein concentrations.

### Statistical analysis

Data shown are mean ± SD. All data were verified in ≥3 independent experiments. Statistical analysis was performed by 1-way ANOVA with Bonferroni post hoc test or Student’s t-test, as stated in figure legends, using PRISM software (GraphPad). Values of P < 0.05 were considered statistically significant.

The datasets generated during and/or analysed during the current study are available from the corresponding author on reasonable request.

### Disclosures

Cees Vermeer is the CSO of VitaK BV, a spin-off company fully owned by Maastricht University. Dina C. Simes and Carla S. B. Viegas are cofounders of GenoGla Diagnostics.

## Electronic supplementary material

Supplemental methods and figures

## References

[1] Hunt JL (2002). Bone formation in carotid plaques: a clinicopathological study. Stroke.

[2] Duer MJ (2008). Mineral surface in calcified plaque is like that of bone: further evidence for regulated mineralization. Arterioscler Thromb Vasc Biol.

[3] Rennenberg RJ (2009). Vascular calcifications as a marker of increased cardiovascular risk: a meta-analysis. Vasc Health Risk Manag.

[4] Sage AP, Tintut Y, Demer LL (2010). Regulatory mechanisms in vascular calcification. Nat Rev Cardiol.

[5] Shroff R, Long DA, Shanahan C (2013). Mechanistic insights into vascular calcification in CKD. J Am Soc Nephrol.

[6] Chatrou ML, Winckers K, Hackeng TM, Reutelingsperger CP, Schurgers LJ (2012). Vascular calcification: the price to pay for anticoagulation therapy with vitamin K-antagonists. Blood reviews.

[7] Shanahan CM, Crouthamel MH, Kapustin A, Giachelli CM (2011). Arterial calcification in chronic kidney disease: key roles for calcium and phosphate. Circ Res.

[8] Jono S (2000). Phosphate regulation of vascular smooth muscle cell calcification. Circ Res.

[9] Schurgers LJ, Uitto J, Reutelingsperger CP (2013). Vitamin K-dependent carboxylation of matrix Gla-protein: a crucial switch to control ectopic mineralization. Trends Mol Med.

[10] Liu Y, Drozdov I, Shroff R, Beltran LE, Shanahan CM (2013). Prelamin A accelerates vascular calcification via activation of the DNA damage response and senescence-associated secretory phenotype in vascular smooth muscle cells. Circ Res.

[11] Neven E, Dauwe S, De Broe ME, D’Haese PC, Persy V (2007). Endochondral bone formation is involved in media calcification in rats and in men. Kidney Int.

[12] Iyemere VP, Proudfoot D, Weissberg PL, Shanahan CM (2006). Vascular smooth muscle cell phenotypic plasticity and the regulation of vascular calcification. J Intern Med.

[13] Frid MG, Shekhonin BV, Koteliansky VE, Glukhova MA (1992). Phenotypic changes of human smooth muscle cells during development: late expression of heavy caldesmon and calponin. Dev Biol.

[14] Huang QQ, Fisher SA, Brozovich FV (1999). Forced expression of essential myosin light chain isoforms demonstrates their role in smooth muscle force production. J Biol Chem.

[15] Feil S, Hofmann F, Feil R (2004). SM22alpha modulates vascular smooth muscle cell phenotype during atherogenesis. Circ Res.

[16] Shankman LS (2015). KLF4-dependent phenotypic modulation of smooth muscle cells has a key role in atherosclerotic plaque pathogenesis. Nat Med.

[17] Alexander MR, Owens GK (2012). Epigenetic control of smooth muscle cell differentiation and phenotypic switching in vascular development and disease. Annu Rev Physiol.

[18] Dhore CR (2001). Differential expression of bone matrix regulatory proteins in human atherosclerotic plaques. Arterioscler Thromb Vasc Biol.

[19] Speer MY (2009). Smooth muscle cells give rise to osteochondrogenic precursors and chondrocytes in calcifying arteries. Circ Res.

[20] Shanahan CM (1999). Medial localization of mineralization-regulating proteins in association with Mönckeberg’s sclerosis evidence for smooth muscle cell–mediated vascular calcification. Circulation.

[21] Li X, Yang HY, Giachelli CM (2008). BMP-2 promotes phosphate uptake, phenotypic modulation, and calcification of human vascular smooth muscle cells. Atherosclerosis.

[22] Giachelli CM (1993). Osteopontin is elevated during neointima formation in rat arteries and is a novel component of human atherosclerotic plaques. J Clin Invest.

[23] Kapustin AN (2015). Vascular smooth muscle cell calcification is mediated by regulated exosome secretion. Circ Res.

[24] Tagariello A (2008). Ucma–A novel secreted factor represents a highly specific marker for distal chondrocytes. Matrix Biol.

[25] Viegas CS (2008). Gla-rich protein (GRP), a new vitamin K-dependent protein identified from sturgeon cartilage and highly conserved in vertebrates. J Biol Chem.

[26] Viegas CS (2009). Gla-rich protein is a novel vitamin K-dependent protein present in serum that accumulates at sites of pathological calcifications. Am J Pathol.

[27] Viegas CS (2015). Gla-rich protein acts as a calcification inhibitor in the human cardiovascular system. Arterioscler Thromb Vasc Biol.

[28] Eitzinger N (2012). Ucma is not necessary for normal development of the mouse skeleton. Bone.

[29] Surmann-Schmitt C (2008). Ucma, a novel secreted cartilage-specific protein with implications in osteogenesis. J Biol Chem.

[30] Lee YJ (2015). Ucma, a direct transcriptional target of Runx2 and Osterix, promotes osteoblast differentiation and nodule formation. Osteoarthritis Cartilage.

[31] Le Jeune M (2010). Identification of four alternatively spliced transcripts of the Ucma/GRP gene, encoding a new Gla-containing protein. Exp Cell Res.

[32] Steitz SA (2001). Smooth muscle cell phenotypic transition associated with calcification: upregulation of Cbfa1 and downregulation of smooth muscle lineage markers. Circ Res.

[33] Hough C, Radu M, Doré JJE (2012). TGF-Beta Induced Erk Phosphorylation of Smad Linker Region Regulates Smad Signaling. PLoS One.

[34] Mizobuchi M, Towler D, Slatopolsky E (2009). Vascular calcification: the killer of patients with chronic kidney disease. Journal of the American Society of Nephrology.

[35] Cheung AK (2000). Atherosclerotic cardiovascular disease risks in chronic hemodialysis patients. Kidney international.

[36] Olechnowicz-Tietz S, Gluba A, Paradowska A, Banach M, Rysz J (2013). The risk of atherosclerosis in patients with chronic kidney disease. International urology and nephrology.

[37] Liu F, Hata A, Baker JC, Doody J (1996). A human Mad protein acting as a BMP-regulated transcriptional activator. Nature.

[38] Heldin C-H, Miyazono K, Ten Dijke P (1997). TGF-β signalling from cell membrane to nucleus through SMAD proteins. Nature.

[39] de Jesus Perez VA (2009). Bone morphogenetic protein 2 induces pulmonary angiogenesis via Wnt–β-catenin and Wnt–RhoA–Rac1 pathways. The Journal of Cell Biology.

[40] Cai T (2016). WNT/beta-catenin signaling promotes VSMCs to osteogenic transdifferentiation and calcification through directly modulating Runx2 gene expression. Exp Cell Res.

[41] Guerrero F (2014). TGF-beta prevents phosphate-induced osteogenesis through inhibition of BMP and Wnt/beta-catenin pathways. PLoS One.

[42] Lin T, Wang X-L, Zettervall SL, Cai Y, Guzman RJ (2017). Dorsomorphin homologue 1, a highly selective small-molecule bone morphogenetic protein inhibitor, suppresses medial artery calcification. Journal of Vascular Surgery.

[43] Proudfoot D, Skepper JN, Shanahan CM, Weissberg PL (1998). Calcification of human vascular cells *in vitro* is correlated with high levels of matrix Gla protein and low levels of osteopontin expression. Arteriosclerosis, thrombosis, and vascular biology.

[44] McCabe KM (2013). Dietary vitamin K and therapeutic warfarin alter the susceptibility to vascular calcification in experimental chronic kidney disease. Kidney international.

[45] Rafael MS (2014). Insights into the association of Gla-rich protein and osteoarthritis, novel splice variants and gamma-carboxylation status. Mol Nutr Food Res.

[46] Shanahan CM, Crouthamel MH, Kapustin A, Giachelli CM (2011). Arterial Calcification in Chronic Kidney Disease: Key Roles for Calcium and Phosphate. Circulation Research.

[47] Willems BA, Vermeer C, Reutelingsperger CP, Schurgers LJ (2014). The realm of vitamin K dependent proteins: shifting from coagulation toward calcification. Mol Nutr Food Res.

[48] Schurgers LJ (2012). Vitamin K-antagonists accelerate atherosclerotic calcification and induce a vulnerable plaque phenotype. PLoS One.

[49] Gregory CA, Gunn WG, Peister A, Prockop DJ (2004). An Alizarin red-based assay of mineralization by adherent cells in culture: comparison with cetylpyridinium chloride extraction. Anal Biochem.

