# Supplementary material for: Ucma/GRP inhibits phosphate-induced vascular smooth muscle cell calcification via SMAD-dependent BMP signalling
Source: Sci Rep. 2018 Mar 21;8:4961. doi: 10.1038/s41598-018-23353-y (PMC5862840; doi:10.1038/s41598-018-23353-y)
Supplement: Supplementary file 1 — Supplemental methods and figures [file 41598_2018_23353_MOESM1_ESM.pdf]

Ucma/GRP inhibits phosphate-induced vascular smooth muscle cell calcification via SMAD-dependent BMP signalling

Brecht A. Willems[1,2,#], Malgorzata Furmanik[1,#], Marjolein M.J. Caron[3], Martijn L.L. Chatrou[1], Dennis HM Kusters[1], Tim J.M. Welting[3], Michael Stock[4], Marta S. Rafael[5], Carla S.B. Viegas[5,6], Dina C. Simes[5,6], Cees Vermeer[2], Chris P.M. Reutelingsperger[1], Leon J. Schurgers[1;*]

1. Department of Biochemistry, School for Cardiovascular Diseases, Maastricht University, Maastricht, The Netherlands
2. VitaK BV, Maastricht University, Maastricht, The Netherlands
3. Department of Orthopedic Surgery, School for Public Health and Primary Care, Maastricht University, Maastricht, The Netherlands
4. Department of Experimental Medicine I, Nikolaus-Fiebiger Centre of Molecular Medicine, University of Erlangen-Nuremberg, Germany
5. Centre of Marine Sciences (CCMAR), University of Algarve, Faro, Portugal
6. GenoGla Diagnostics, Centre of Marine Sciences (CCMAR), University of Algarve, Faro, Portugal

\# These authors contributed equally.

* Corresponding author:

Leon J. Schurgers, PhD

Department of Biochemistry, Maastricht University

PO Box 616, 6200 MD Maastricht

The Netherlands

T: 31 43 3881680 // F: 31 43 388 4159

E: l.schurgers@maastrichtuniversity.nl

Supplemental methods

SiRNA transfections

10000 human VSMCs were seeded per well of a 48-well plate in M199 (Gibco) supplemented with 20% FBS, peniciliin and streptomycin. 24 hours later cells were transfected using 3 µl HiPerfect reagent (Qiagen, Venlo, the Netherlands) with 1.5 µmol Ucma/GRP siRNA or a scramble control (Qiagen, Venlo, the Netherlands) per well. After 48 hours osteogenic medium was applied and calcification assays carried out as described in the main article.

Boyden Chamber experiments

20000 Ucma/GRP$^{-/-}$ or WT VSMCs were seeded per well into the upper or lower chamber and allowed to attach overnight. The next day, cells were treated with control or osteogenic medium. After incubating the cells for 12 days, calcification of cells in lower chambers was quantified and normalized to protein content.

Matrigels – extracellular vesicle-induced calcification

For coating plates, matrigel solution (20 mg/ml) was added to each well and incubated for 1 hour. 20000 Ucma/GRP$^{-/-}$ or WT cells were seeded into the upper wells of a Boyden chamber and allowed to attach overnight. Next, the upper wells were placed in the matrigel coated Boyden chambers. Calcification medium was added and after 12 days. 0.1M NaOH with 0.1% SDS were added to matrigels to obtain protein lysates. Calcification was normalised to protein content to correct for the amount of vesicles in the medium.

Conditioned medium experiments

20000 Ucma/GRP$^{-/-}$ or WT VSMCs were seeded per well of 24-well plate (Corning, Amsterdam, the Netherlands). The next day, cells were treated with control, osteogenic or conditioned medium for 12 days and calcification was measured. Medium was conditioned by incubating cells for 7 days in serum

free growth medium (DMEM supplemented with penicillin and streptomycin). After harvesting, medium was centrifuged for 5 minutes at 2000 rpm (Rotina 380R, Hettich, Geldermalsen, the Netherlands), filtered through a 0.22µm filter and stored at 4°C. FCS was added before the conditioned medium was applied to cells.

Solid phase binding assays

ELISA plates were coated with BSA (used as blank) and the RP-HPLC purified proteins cGRP (sturgeon GRP) (1), ucGRP (recombinant non-carboxylated human GRP produced in *E. coli*) (2) andbovine MGP (2) (used as positive control), and recombinant BMP-2 (Preprotech), all at 0,5µg/well. After blocking with BSA, plates were incubated with BMP-2 (500 ng/ml). Bound BMP-2 was detected using BMP2/4 monoclonal antibody (Santa Cruz Biotechnology, sc-137087, 1:100) and anti-mouse-IgG-HRP antibody (Sigma). TMB (3,3',5,5' tetramethylbenzidine, Sigma) was used as a substrate and colorimetric detection was performed at 450 nm.

Supplemental figures

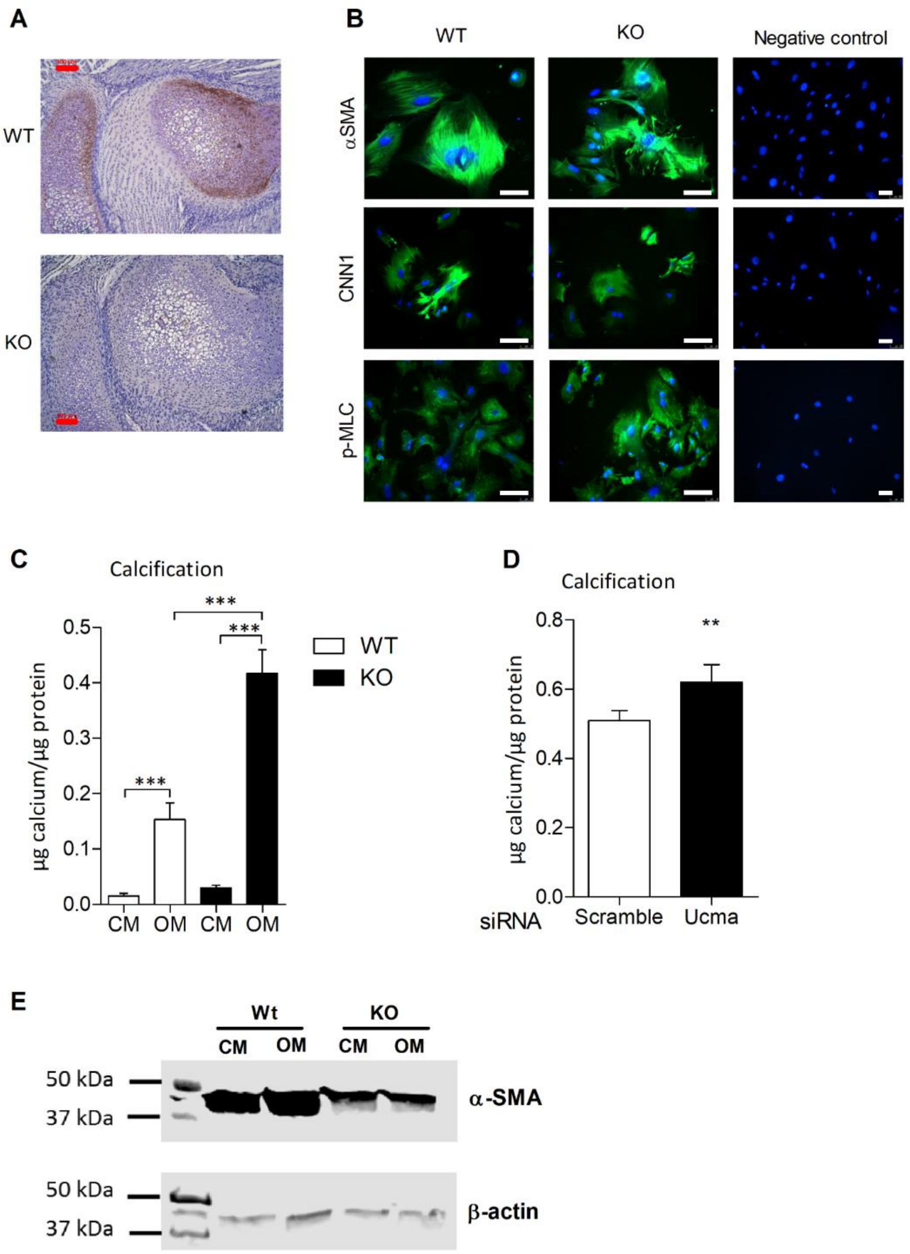

Supplemental Figure 1. **A.** Immunohistochemical staining of Ucma/GRP in epiphysae of WT and KO mice confirmed the absence of Ucma/GRP in KO mice**.** Scale bars are 100 µm**. B.** Immunostaining of contractile markers in VSMCs isolated from WT and Ucma/GRP$^{-/-}$ mice to confirm the identity of cells. Scale bars are 100 µm. Negative control – cells incubated with secondary antibody only. **C.** mVSMCs were treated with control growth medium (CM) and osteogenic medium (OM, with 2.6mM phosphate) and calcification was quantified after 12 days. N=4-6, statistical significance was assessed using t-test. **D.** Calcification was measured in WT transfected with Ucma/GRP siRNA and treated with osteogenic medium (2.6mM phosphate) for 12 days. N=4, statistical significance was assessed using t-test. **E.** Western blotting analysis of αSMA expression in Ucma/GRP$^{-/-}$ and WT cells treated with control growth medium (CM) and osteogenic medium (OM, with 2.6mM phosphate) after 12 days. All graphs show mean+SD. **p<0.001-0.01, ***p<0.001.

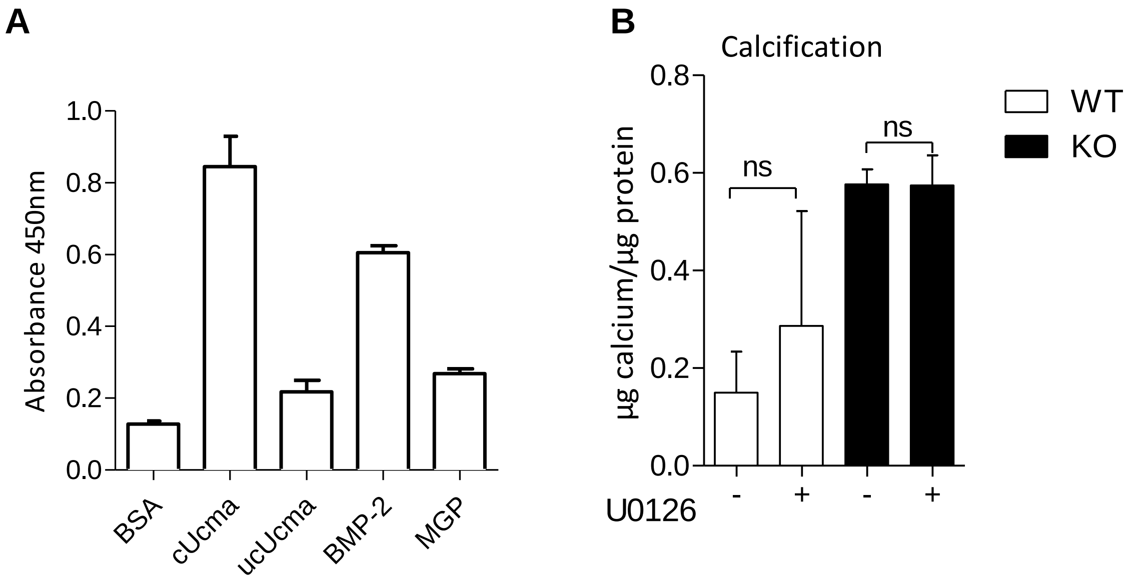

Supplemental Figure 2. A. ELISA plates were coated with BSA (negative control), carboxylated Ucma/GRP (cUcma), uncarboxylated Ucma/GRP (ucUcma), BMP-2 and MGP (positive control), 0.5 µg/well each. After blocking with BSA, plates were incubated with BMP-2 (500 ng/ml). Bound BMP-2 was detected using BMP-2/4 antibody, anti-mouse-IgG-HRP antibody, and TMB-based colorimetric detection at 450 nm. N=2, graph shows mean+SD. **B.** MVSMCs from Ucma/GRP$^{-/-}$ and WT mice were treated with an inhibitor of Erk phosphorylation (U0126, 2µM) in the presence of osteogenic medium (OM, with 2.6mM phosphate) for 12 days, after which calcification was quantified. N=8, graph shows mean+SD. Statistical significance was assessed using ANOVA.

References

1. Viegas CS, Simes DC, Laize V, Williamson MK, Price PA, Cancela ML. Gla-rich protein (GRP), a new vitamin K-dependent protein identified from sturgeon cartilage and highly conserved in vertebrates. J Biol Chem. 2008;283(52):36655-64.
2. Viegas CS, Herfs M, Rafael MS, Enriquez JL, Teixeira A, Luis IM, et al. Gla-rich protein is a potential new vitamin K target in cancer: evidences for a direct GRP-mineral interaction. Biomed Res Int. 2014;2014:340216.
